# *KCNT2*-Related Disorders: Phenotypes, Functional, and Pharmacological Properties

**DOI:** 10.1002/ana.26662

**Published:** 2023-05-22

**Authors:** Maria Cristina Cioclu, Ilaria Mosca, Paolo Ambrosino, Deborah Puzo, Allan Bayat, Saskia B. Wortmann, Johannes Koch, Vincent Strehlow, Kentaro Shirai, Naomichi Matsumoto, Stephan J. Sanders, Vincent Michaud, Marine Legendre, Antonella Riva, Pasquale Striano, Hiltrud Muhle, Manuela Pendziwiat, Gaetan Lesca, Giuseppe Donato Mangano, Rosaria Nardello, KCNT2-study group, Johannes R. Lemke, Rikke S. Møller, Maria Virginia Soldovieri, Guido Rubboli, Maurizio Taglialatela

**Affiliations:** 1 Department of Epilepsy Genetics and Personalized Medicine (member of ERN EpiCARE), Danish Epilepsy Centre, Dianalund, Denmark; 2 Department of Biomedical, Metabolic, and Neural Science, University of Modena and Reggio Emilia, Modena, Italy; 3 Department of Medicine and Health Sciences “Vincenzo Tiberio”, University of Molise, Campobasso, Italy; 4 Dept. of Science and Technology, University of Sannio, Benevento, Italy; 5 Department of Regional Health Research, University of Southern Denmark, Odense, Denmark; 6 University Children’s Hospital, Paracelsus Medical University, Salzburg, Austria; 7 Amalia Children’s Hospital, Nijmegen, The Netherlands; 8 Institute of Human Genetics, University of Leipzig Medical Center, Leipzig, Germany; 9 Department of Pediatrics, Tsuchiura Kyodo General Hospital, Tsuchiura, Japan; 10 Department of Human Genetics, Yokohama City University Graduate School of Medicine, Yokohama, Japan; 11 Department of Psychiatry and Behavioral Sciences, UCSF Weill Institute for Neurosciences, University of California, San Francisco, CA, USA; 12 Institute for Human Genetics, University of California, San Francisco, CA, USA; 13 Bakar Computational Health Sciences Institute, University of California, San Francisco, CA, USA; 14 Service de Génétique Médicale, Centre de Référence Anomalies du Développement et Syndrome Malformatifs, Centre Hospitalier Universitaire de Bordeaux, Bordeaux, France; 15 Maladies rares: Génétique et Métabolisme (MRGM), INSERM U1211, Université de Bordeaux, Bordeaux, France; 16 IRCCS Istituto Giannina Gaslini, Genoa, Italy; 17 Department of Neurosciences, Rehabilitation, Ophthalmology, Genetics, Maternal and Child Health, University of Genoa, Genoa, Italy; 18 Department of Neuropediatrics, University Medical Centre Schleswig-Holstein, Christian-Albrechts-University, Kiel, Germany; 19 Institute of Clinical Molecular Biology, Christian-Albrechts-University of Kiel, Kiel, Germany; 20 Pathophysiology and Genetics of Neuron and Muscle (PNMG), UCBL, CNRS UMR5261—INSERM U1315, Lyon, France; 21 Department of Medical Genetics, University Hospital of Lyon and Claude Bernard Lyon I University, Lyon, France; 22 Department of Biomedicine, Neuroscience and Advanced Diagnostics, University of Palermo, Palermo, Italy; 23 Department of Health Promotion, Mother and Child Care, Internal Medicine and Medical Specialties “G. D’Alessandro”, University of Palermo, Palermo, Italy; 24 Center for Rare Diseases, University of Leipzig Medical Center, Leipzig, Germany; 25 University of Copenhagen, Copenhagen, Denmark; 26 Department of Neuroscience, University of Naples “Federico II”, Naples, Italy

## Abstract

**Objective:**

Pathogenic variants in *KCNT2* are rare causes of developmental epileptic encephalopathy (DEE). We herein describe the phenotypic and genetic features of patients with *KCNT2*-related DEE, and the *in vitro* functional and pharmacological properties of KCNT2 channels carrying 14 novel or previously untested variants.

**Methods:**

Twenty-five patients harboring *KCNT2* variants were investigated: 12 were identified through an international collaborative network, 13 were retrieved from the literature. Clinical data were collected and included in a standardized phenotyping sheet. Novel variants were detected using exome sequencing and classified using ACMG criteria. Functional and pharmacological studies were performed by whole-cell electrophysiology in HEK-293 and SH-SY5Y cells.

**Results:**

The phenotypic spectrum encompassed: (a) intellectual disability/developmental delay (21/22 individuals with available information), ranging from mild to severe/profound; (b) epilepsy (15/25); (c) neurological impairment, with altered muscle tone (14/22); (d) dysmorphisms (13/20). Nineteen pathogenic *KCNT2* variants were found (9 new, 10 reported previously): 16 missense, 1 in-frame deletion of a single amino acid, 1 nonsense, and 1 frameshift. Among tested variants, 8 showed gain-of-function (GoF), and 6 loss-of-function (LoF) features when expressed heterologously *in vitro*. Quinidine and fluoxetine blocked all GoF variants, whereas loxapine and riluzole activated some LoF variants while blocking others.

**Interpretation:**

We expanded the phenotypic and genotypic spectrum of *KCNT2*-related disorders, highlighting novel genotype–phenotype associations. Pathogenic *KCNT2* variants cause GoF or LoF *in vitro* phenotypes, and each shows a unique pharmacological profile, suggesting the need for in vitro functional and pharmacological investigation to enable targeted therapies based on the molecular phenotype.

*KCNT2* encodes K_Na_1.2 (also known as Slo2.1 or “Slick”, Sequence like an intermediate conductance K^+^ channel) subunits belonging to the Slo family of potassium (K^+^) channels. Slo channels are mainly regulated by Ca^2+^, Na^+^, and Cl^−^ ions, and assemble as tetramers of identical or compatible subunits, each containing 6 membrane-spanning domains (from S_1_ to S_6_) clustered around a water-filled, K^+^-selective pore.^1,2^ They are widely distributed in the brain, where they play an important role in the adaptation of neuronal firing patterns and generation of the slow after-hyperpolarization following repetitive trains of action potentials.^3,4^

Numerous pathogenic variants in *KCNT1*, which encodes for K_Na_1.1 subunits highly-homologous to K_Na_1.2, are associated with a spectrum of epileptic conditions including epilepsy of infancy with migrating focal seizures (EIMFS) and autosomal dominant nocturnal frontal lobe epilepsy.^5,6^ With a single exception,^7^ all *KCNT1* pathogenic variants display gain-of-function (GoF) properties.^8^ Repurposed quinidine,^9^ newly identified small-molecules,^10,11^ or ASO-based gene silencing^12^ have been proposed as precision medicine approaches for these patients.

In contrast, only few *KCNT2* variants have been reported in a limited number of patients with developmental and epileptic encephalopathy (DEE) or neurodevelopmental disorders.^13–20^ In vitro functional characterization of some variants in heterologous expression systems has demonstrated change-of-function, GoF or loss-of-function (LoF) properties.^14,16,19^ At present, no genotype–phenotype correlation has been identified and no comprehensive pharmacological study has been carried out.

Herein, we describe the genotypes and phenotypes of 25 (12 novel and 13 previously reported) patients harboring *KCNT2* variants. To highlight possible correlations between genotypes, consequences on channel activity, and clinical phenotypes, in vitro electrophysiological investigations were performed in *KCNT2* channels carrying all newly-found and previously-reported but not yet characterized variants. In addition, to identify targeted treatments based on the ability of repurposed agents to correct the functional changes observed *in vitro*, pharmacological studies using selected potassium channel blockers or openers were also carried out.

## Subjects/Materials and Methods

### Unpublished Patients—Ascertainment and Phenotyping

Novel patients were collected through an international network of Epilepsy and Genetic Centers. Clinical data were collected via a standardized phenotyping sheet, and seizures, classified according to the 2017 International League Against Epilepsy proposal,^21^ were assigned, whenever possible, to defined epileptic syndromes. EEG interpretation, seizure outcome, intellectual disability (ID), developmental delay (DD), and autism spectrum disorder (ASD) were assessed by the referring physicians. ID/DD was classified as none, mild, moderate, severe, and profound. Formal neuropsychological assessments were available in 3 patients. Informed written consent from parents or caregivers and approval from local ethics committees were obtained.

### Literature Review

A PubMed search was performed using the term “KCNT2” (latest update 14/05/2022) limited to English language. We identified 8 publications reporting 13 patients; additional clinical information regarding 2 previously reported patients (#17 and #21, Supplementary Table S2) were collected upon contacting the authors of the respective publication.

### Genotyping

In newly identified patients, variants were detected using whole-exome sequencing and validated through Sanger sequencing. Patients were referred for genetic testing because of early-onset epilepsy and/or neurodevelopmental disorders. Family segregation studies were performed from DNA obtained from blood samples in 10/12 patients; parents were unavailable for testing in individuals #1 and #6. Variants were classified as pathogenic or likely pathogenic according to the ACMG criteria.^22^ None of the variants identified in the novel cohort was found in the Genome Aggregation Database (gnomAD, https://gnomad.broadinstitute.org/).^23^ In addition, we analyzed 3 *KCNT2* variants considered of uncertain significance (VUS); 2 of those (R47K and L313S) were absent from gnomAD, whereas the T910M allele is reported in 3 individuals in gnomAD.

### Functional Analysis

#### Mutagenesis and Transfection.

Mutations were obtained through Quick-change Site-Directed Mutagenesis in a plasmid encoding for a tGFP tagged isoform (NM_198503) of human KCNT2 subunits (RG216225; Origene, Rockville, MD) and verified by Sanger sequencing. Wild-type and/or mutant KCNT2 cDNAs were transiently transfected in HEK-293 (Human Embryonic Kidney)^14,24^ or in SH-SY5Y human neuroblastoma cells differentiated by a 5 day exposure to 10 μM retinoic acid (RA), as described.^25^ A plasmid encoding the enhanced green fluorescent protein (eGFP) was used as a transfection marker.

#### Electrophysiological and Pharmacological Studies.

Macroscopic currents were recorded at room temperature from HEK-293 or RA-differentiated SH-SY5Y cells 24 or 48 hours after transfection, respectively, using the patchclamp technique in the whole-cell configuration, with an Axopatch 200B amplifier (Molecular Devices, Union City, CA) and pCLAMP10 software (Axon Instruments). Pipette (intracellular, without ATP) and extracellular solution composition, as well as methods for data acquisition and analysis were previously described.^14,24^ Drugs (Sigma-Aldrich, Milan, Italy) were dissolved in chloroform (quinidine; final vehicle concentration 0.01%) or DMSO (fluoxetine, loxapine, riluzole; final vehicle concentration 0.1–0.01%), and perfused using a fast solution exchange system.

#### Molecular Modeling.

Closed (PDB 5U76) and open (PDB 5U70) configurations of chicken KCNT1^26^ served as templates for homology models of human KCNT2.^14^ The models were then analyzed by using Discovery Studio 4.0 Client software (BIOVIA, San Diego, CA, USA).

#### Statistics.

Data are expressed as mean ± standard error of the mean (SEM). Statistically significant differences were evaluated with the Student’s t-test or with analysis of variance (ANOVA) followed by the Student–Newman–Keuls test (threshold: *p* < 0.05).

## Results

We identified 25 patients (13 males and 12 females) with pathogenic variants in *KCNT2*, with an age range of 3 months-40 years (median 9 years): 12 patients are unpublished (Tables 1 and 2), whereas 13 have been previously reported (Supplementary Table S2). Denominators indicate the number of patients in whom the information reported is available. Clinical, demographic, genetic, and neuroradiological features of patients 13–25, previously published, are summarized in Supplementary Table S2.

### Genetic Findings

**FIGURE 1:**
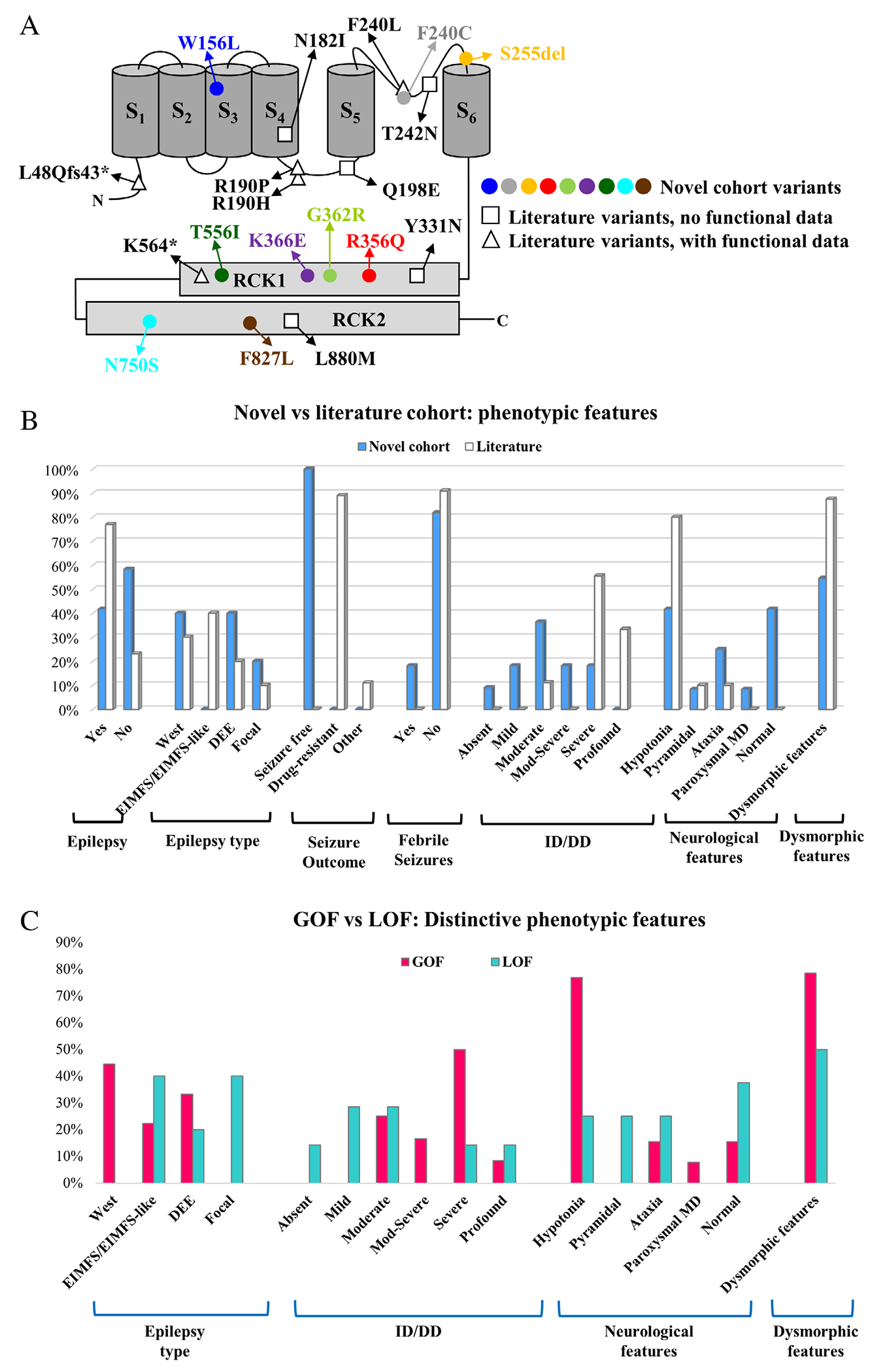
Localization of the variants and distinctive phenotypic features. (A) Schematic topology of a KCNT2 subunit and localization of the variants identified in the novel cohort (colored circles), the ones already described in literature but not functionally characterized (empty squares), or the already-described and functionally characterized (empty triangles) variants. (B) Representation of the main phenotypic features in the novel cohort (light blue bars) versus the literature cohort (white bars); values are expressed as the percentage of patients presenting the trait out of the number of patients in whom the information is available. (C) Representation of the main phenotypic features in patients carrying GoF (red bars) versus LoF variants (green bars); values are expressed as the percentage of patients presenting the trait out of the number of patients in whom the information is available.

Trio-offspring sequencing data, available for 21 patients, revealed a de novo heterozygous *KCNT2* variant in 19 patients (Table 1). Patient #2 inherited the variant from the asymptomatic mosaic father (15% somatic mosaicism), while patient #5 inherited the variant from her mildly affected mother (patient #6). Overall, 19 pathogenic *KCNT2* variants were found, 9 in the novel cohort and 10 in previously reported patients; of these, 16 are missense, 1 is an in-frame deletion of a single amino acid (S255del), 1 is a nonsense (K564*), and 1 is a frameshift (L48Qfs43*) variant. The only recurrent variants were observed at the p190 position (R190H in patients #2, #3, #15, #16, #17; R190P in patients #18 and #19).^14,18,20^ Figure 1A depicts the location of each variant in a schematic representation of a KCNT2 subunit.

### Phenotypic Features

#### Neurodevelopmental and Behavioral Phenotypes.

ID/DD was profound in 3/20 patients (15.00%) (15%), severe or moderate–severe in 9/20 patients (45.00%) (45%), moderate in 5/20 (25%), mild in patients #1 and #6 (10.00%) (10%), absent in patient #8 (Table 1; Supplementary Table S2). Two patients (#13 and #25) were reported as delayed without further details, whereas for 3 patients the degree of ID/DD was not inferable due to young age (#7, #23 and #24).

The median age at walking was 25 months (range 9–60 months). Patient #21 started to walk with assistance at the age of 4 years and at the current age of 10 years he is wheelchair-bound. Patient #7 started crawling at 21 months of age, while patient #14 was still unable to walk at 6 years of age. In 4 patients, these data were missing, and patients #23 and #24 were too young to be assessed.

Patients #1 and #6, despite featuring a mild ID/DD, exhibited a significant language delay. Individual #8 displayed normal developmental milestones and language acquisition; formal neuropsychological testing showed an IQ score of 97. Of the remaining patients older than 5 years, 12/17 were able to speak only few words or simple sentences and 5/17 were non-verbal (Table 1; Supplementary Table S2). Patient #10 showed a normal initial acquisition of motor and language skills, with a significant slowdown after the age of 11 months.

Seven/seventeen (41.18%) patients were diagnosed with autism spectrum disorders (ASD). Behavioral disorders, mainly hyperactivity, attention deficit and aggressiveness, were observed in 11/15 (73.33%) patients (Table 1; Supplementary Table S2).

#### Neurological Findings.

Hypotonia was observed in 13/22 (59.09%) patients. A mild symmetrical bilateral weakness was found in patient #9. Ataxia was reported in patients #9, #10, #11, and #22. Patient #2 presented with brief (<1 min) episodes of paroxysmal dystonia triggered by fever occurring up to 50 times/day from the age of 8 months until spontaneous remission at age 4 years. In 5 patients (#4, #5, #6, #8, #12), neurological examination was unremarkable.

#### Dysmorphic Features.

Thirteen/Twenty (65.00%) (65%) patients showed dysmorphisms. Eleven exhibited similar features including pronounced curved eyebrows, thick eyelashes, broad nasal tip, hypertrichosis, diastema (Supplementary Figure S1); patient #5 presented hypertelorism, short down slanted palpebral fissures, arched eyebrows, flat nasal root, bulbous nose tip, anteverted nares, brachydactyly, clinodactyly of the fifth finger, syndactyly of second and third toes, flat feet whereas patient #12 showed simplified auricles and camptodactyly with clinodactyly of the third fingers.

#### Other Features.

Sleep disorders, such as parasomnias and frequent nocturnal awakenings, responding to melatonin, were described in 5/11 patients. Patients #4 and #11 presented acquired microcephaly. No cardiovascular disorders were reported in literature nor in our novel cohort of patients. Peripheral nervous system involvement with low amplitude distal motor responses in lower extremities recorded in neurophysiological studies has been reported in only one patient (#14). Additional features are reported in Table 1 and in Supplementary Table S2.

#### Epilepsy.

Fifteen/Twenty-five patients suffered from epilepsy: 5/12 (41.67%) patients in the novel cohort (Table 2) and 10/13 (77%) patients in the literature cohort (Supplementary Table S2). The median age at seizure onset was 4 months, with a significant difference between the literature cohort (median 2.5 months, range 1 day-8 months) and the novel cohort (median 6 months, range 1–14 months).

West syndrome (WS) was diagnosed in 5 patients (#3, #7, #15, #21, #23); in patient #7 multiple daily clonic and myoclonic seizures followed spasms offset whereas in patient #23 the spasms were preceded by Ohtahara syndrome. Four patients displayed electroclinical features consistent with EIMFS or EIMFS-like developmental and epileptic encephalopathy (DEE) (#13, #18, #20, #24). Patient #1 had focal epilepsy with motor seizures with or without bilateral tonic–clonic evolution, triggered by fever, while patient #22 experienced hyperkinetic focal motor seizures, preceded by tonic seizures at onset; patient #4, diagnosed with developmental encephalopathy, presented 2 tonic–clonic seizures and a seizure cluster between the first and fourth month of life with subsequent seizure freedom. Patient #11 had febrile seizures at 6 months of age; he subsequently presented febrile and afebrile bilateral tonic–clonic seizures also experiencing an episode of convulsive status epilepticus. Two patients (#14 and #25) were reported to have an unspecified DEE.

**FIGURE 2:**
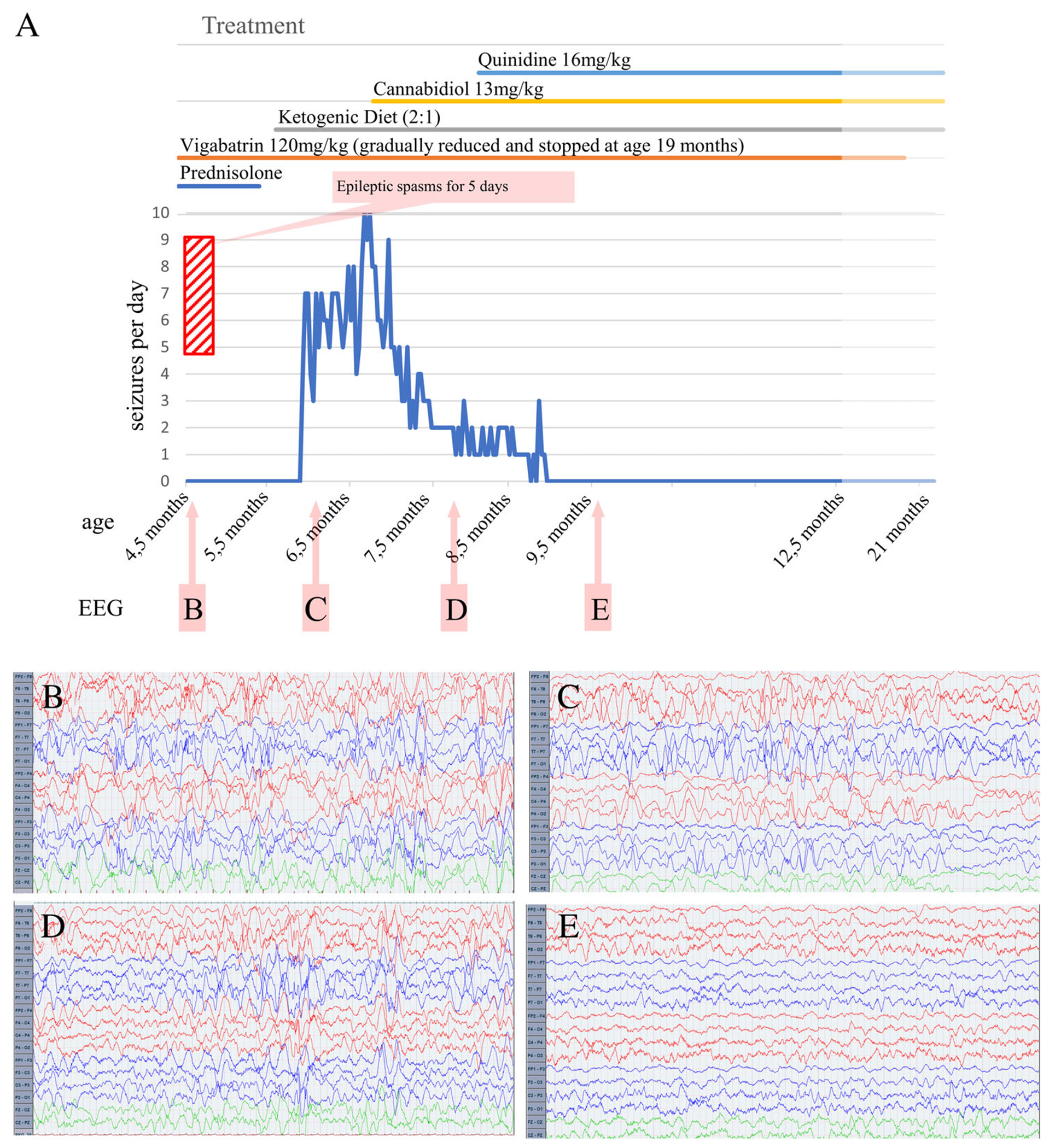
Clinical and EEG evolution in patient #7 following treatment changes. (A) Graphical representation of the changes of seizure frequency following the modifications of treatments indicated on top of the panel. The vertical red hatched column indicates a period of 5 days during which the child had an increase of spasms. The introduction of cannabidiol (CBD) during treatment with vigabatrin (VGB) and ketogenic diet (KD) was associated with a decrease in seizure frequency. The seizures gradually disappeared upon introduction of quinidine. (B), (C), (D), and E) are the time points where the EEG (panels below) were recorded. (B) EEG showed hypsarrhythmia during treatment with VGB; (C) hypsarrhythmia persisted with bursts of fast activities superimposed under treatment with VGB associated with KD. (D) Disappearance of hypsarrhythmia and persistence of sporadic high amplitude epileptiform discharges after the introduction of CBD to KD and VGB; (E) further improvement of the EEG with disappearance of epileptiform discharges after the introduction of quinidine associated with CBD, KD, and VGB. [Color figure can be viewed at www.annalsofneurology.org ]

At last follow-up (mean duration: 56.6 months, range: 6 months-13 years) all 5 novel patients with epilepsy were seizure-free. Three/five were still taking antiseizure medications (ASM); patient #3 was weaned off medication at the age of 5 years. Seizure freedom was achieved with valproate (patient #1), with phenobarbital (patient #4), with the combination of quinidine, ketogenic diet (KD), and cannabidiol (#7, Fig. 2), or with sulthiame and KD (#11).

In the literature cohort, 8/9 patients were refractory to various combinations of ASMs while patient #18 stopped taking phenobarbital after the disappearance of tonic–clonic seizures, despite the persistence of absences. Two patients were started on ketogenic diet (#15 and #21) and in one of them (#15) quinidine was tried as well, with temporary positive effects; however further increase of quinidine dosing was not attempted due to QTc prolongation.^14^

#### Electroencephalography and Neuroimaging.

The most common EEG abnormalities were multifocal epileptic discharges (8/18, 44.44% patients), hypsarrhythmia (5/18, 27.78%), and focal epileptic activity (2/18, 11.11%). Interestingly, EEG was abnormal also in 3 patients without epilepsy (#9, #10, #17). Hypsarrhythmia later evolved to diffuse poly-spike waves or intermittent sharp-slow waves and general slowing; in patient #23 this EEG picture was preceded by a burst suppression pattern. Brain MRI showed mild abnormal findings in only 5/17 (29.41%) patients, whereas it was normal in the remaining cases (Table 2; Supplementary Table S2).

### Functional Analysis of KCNT2 Channels Carrying Disease-Causing Variants

**FIGURE 3:**
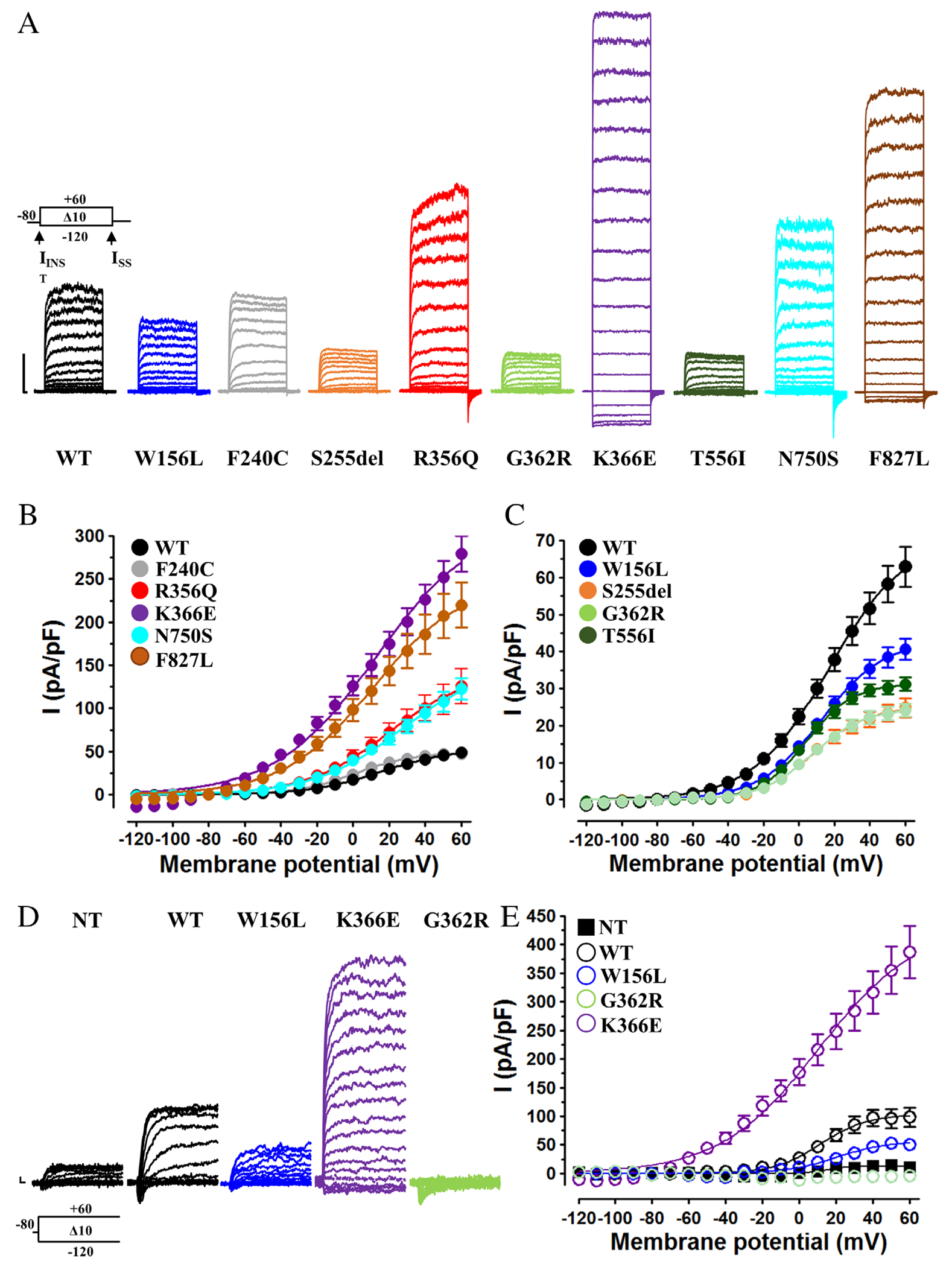
Biophysical properties of KCNT2 homomeric channels incorporating newly-identified variants. (A) Representative whole-cell currents traces recorded from HEK cells expressing the indicated channels upon exposure to the protocol shown above the leftmost group of traces. Current scale: 1 nA; time scale: 20 msec. (B, C) Average current densities from cells expressing the indicated channels incorporating GoF (B) or LoF (C) variants. (D, E) Representative whole-cell current traces (D) and average current densities (E) recorded from RA-differentiated SH-SY5Y expressing the indicated channels upon exposure to the protocol shown below the leftmost group of traces. Current scale: 200 pA; time scale: 2 msec.

Among the 12 newly identified patients, 2 (#2 and #3) carried the R190H variant (Table 1), a recurrent variant whose functional properties have been previously described.^14,18^ This variant, as well as the R190P, affecting the same residue (previously described in patients #18 and #19), displayed in vitro functional features consistent with GoF effects.^14^ The functional features of the 9 new variants found in the remaining patients are reported in Fig. 3.

Wild-type KCNT2 currents activated at membrane voltages > − 40 mV, showing strong outward rectification.^27^ The activation process of KCNT2 current at +40 mV (I_STEADY-STATE_ or I_SS_) was described by 2 distinct components: an instantaneous voltage-independent one (I_INST_), accounting for about 50% of the total current, followed by a slower voltage- and time-dependent component (I_vdep_). Table 3 reports the biophysical features (midpoint potential of activation, V_1/2_; slope factor, *k*; I_INST_/I_SS_ ratio) of the currents carried by all experimental groups investigated. When compared to wild-type KCNT2, homomeric KCNT2 channels carrying the R356Q, K366E, N750S, or F827L variants generated outwardly rectifying currents whose densities were significantly larger, a functional feature consistent with a GoF in vitro phenotype. In addition, KCNT2 channels with F240C, R356Q, K366E, or F827L, but not N750S, variants displayed a marked hyperpolarizing shift in V_1/2_; finally, KCNT2 channels with K366E or F827L showed an increase in the relative size of the I_INST_/I_SS_ ratio and a shallower slope of the G/V relationship. By contrast, homomeric KCNT2 channels carrying the W156L, S225del, G362R, or T556I variants generated outwardly rectifying currents whose densities were smaller than those of wild-type KCNT2 channels, a functional feature indicative of a LoF *in vitro* phenotype. In addition, KCNT2 T556I currents also showed a significant decrease in the I_INST_/I_SS_ ratio, with no changes in V_1/2_ or *k* (Table 3).

To investigate whether the functional alterations described in HEK-293 cells also occurred in a neuronal cellular context, KCNT2 channels (wild-type and selected variants) were expressed in RA-differentiated human neuroblastoma SH-SY5Y cells, a widely used neuronal cellular model (Fig. 3D,E).^28^ In these cells, transient transfection of KCNT2 channels generated outward currents whose density was significantly larger than those from nontransfected cells (n = 6–10; *p* < 0.05). In addition, similarly to HEK-293 cells, when compared to wild-type KCNT2, current density was significantly increased upon expression of KCNT2 K366E channels (n = 13; *p* < 0.05 versus WT) and decreased when KCNT2 W156L or G362R channels were expressed (n = 5–14; *p* < 0.05 versus WT). These results suggested that these variants led to qualitatively similar functional changes (GoF or LoF effects, respectively) when expressed in both non-neuronal HEK-293 or neuron-like SH-SY5Y cells.

Additional experiments were also carried out in HEK-293 cells to investigate the *in vitro* properties of all 5 previously identified but not functionally characterized pathogenic *KCNT2* variants (Fig. 1A). These include the N182I and L880M variants recently found in 2 unrelated patients with neurodevelopmental disorders,^13^ the T242N variant found in a patient with DEE characterized by drug-resistant hyperkinetic frontal focal seizures,^17^ and the Q198E and Y331N variants described in 2 unrelated patients diagnosed with DEE characterized by profound developmental delay and intractable infantile-onset seizures.^15^ When compared to wild-type KCNT2 currents, among these 5 variants, 3 (Q198E, Y331N, and L880M) showed GoF functional features in vitro consisting of an increased current density (Q198E, Y331N, and L880M), accompanied by a hyperpolarizing shift in V_1/2_ (Q198E, Y331N, and L880M), and an increased I_INST_/I_SS_ ratio (Q198E only). Instead, KCNT2 channels carrying the N182I and T242N variants displayed a reduced current density, with KCNT2 T242N channels also showing reduced relative size of the I_INST_/I_SS_ ratio; both these functional features are consistent with a LoF *in vitro* phenotype. Altogether, these data indicate that, at variance with *KCNT1* whose pathogenic variants are almost invariably GoF,^29^ *KCNT2* variants with either GoF or LoF in vitro phenotypes might be causative of severe neurodevelopmental disorders.

Finally, the functional features of KCNT2 channels carrying the 3 VUS variants investigated (R47K, L313S, and T910M) were undistinguishable from those of wild-type KCNT2 channels (Table 3). Therefore, no indication for their potential pathogenic role was found, and we considered these 3 VUS to be less likely disease-associated, although they formally remained VUS.

### Molecular Modeling

**FIGURE 4:**
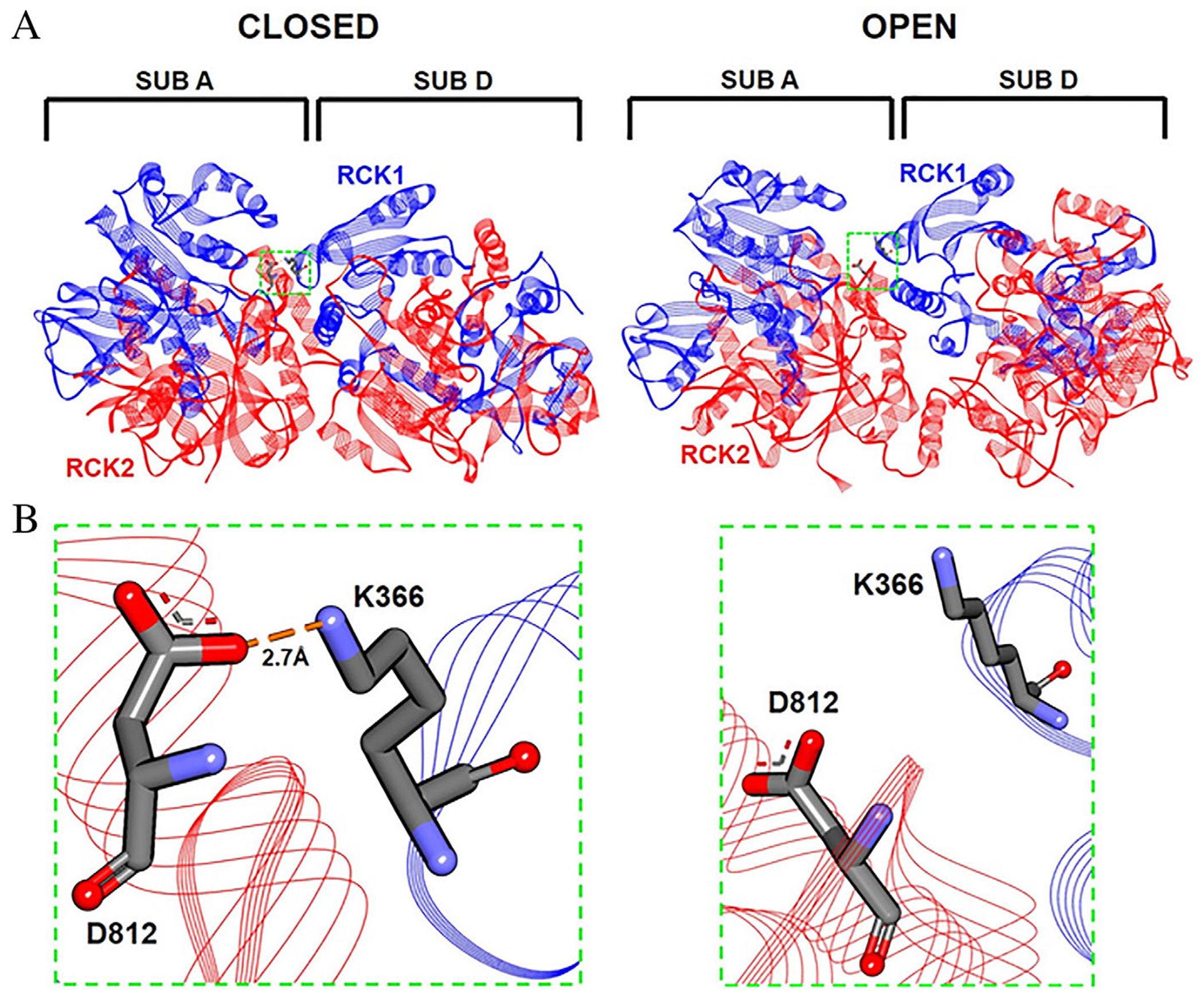
Structural analysis of K366 interactions. (A) Left panel: 3D-structures of the A and D subunits of a KCNT2 channel in the closed state; B and C subunits were removed for clarity. The green box identifies the protein region including the K366 residue. Right panel enlarged view of the region encompassing the K366 and D182 residues. The orange line indicates an electrostatic interaction occurring between K336 and D812 residues. (B) Left panel: 3D-structures of the A and D subunits of KCNT2 channels in the open state; B and C subunits were removed for clarity. The green box identifies the protein region including the K366 residue. Right panel enlarged image of the region encompassing the K366 and D182 residues. No electrostatic interaction occurs between K336 and D812 residues in the open state.

Starting from the inactive (closed) and activated (open) conformations of chicken KCNT1 channels,^26^ state-dependent homology modeling was performed to infer potential mechanism(s) by which some of the KCNT2 residues affected by the herein described mutations might regulate channel opening. Supplementary Table S3 describes the intra- and inter-subunit interactions identified by this analysis when each of the amino acid positions affected by the missense mutations are occupied by the wild-type (top panel) or by the mutant residues (bottom panel). In most LoF variants, changes mostly involved interactions occurring in the open state, in terms of total number of interactions (N182I) and/or type of residues involved (N182I). By contrast, for channels carrying GoF variants, a reduced number of interactions was revealed in the closed state (ie, for F240C, R356Q, and L880M); in GoF variants, an increased number of open state interactions was also revealed (ie, for Q198E, F827L, and L880M). In particular, in the tetrameric structure of KCNT2 channels, the K366 residue in the RCK1 domain is involved in a state-dependent ionized hydrogen bond with the D812 residue of a neighboring subunit. This interaction only occurs in the closed state (Fig. 4), suggesting a preferential contribution in stabilizing the resting (closed) state of the channel. Replacement of the lysine residue at position 366 with glutamate (K366E) abolishes the interaction of this residue with the D812 residue, likely promoting constitutive channel opening and explaining the strong GoF in vitro phenotype prompted by the K366E variant.

### In Vitro Pharmacology of KCNT2 Channels Carrying Disease-Causing Variants

**FIGURE 5:**
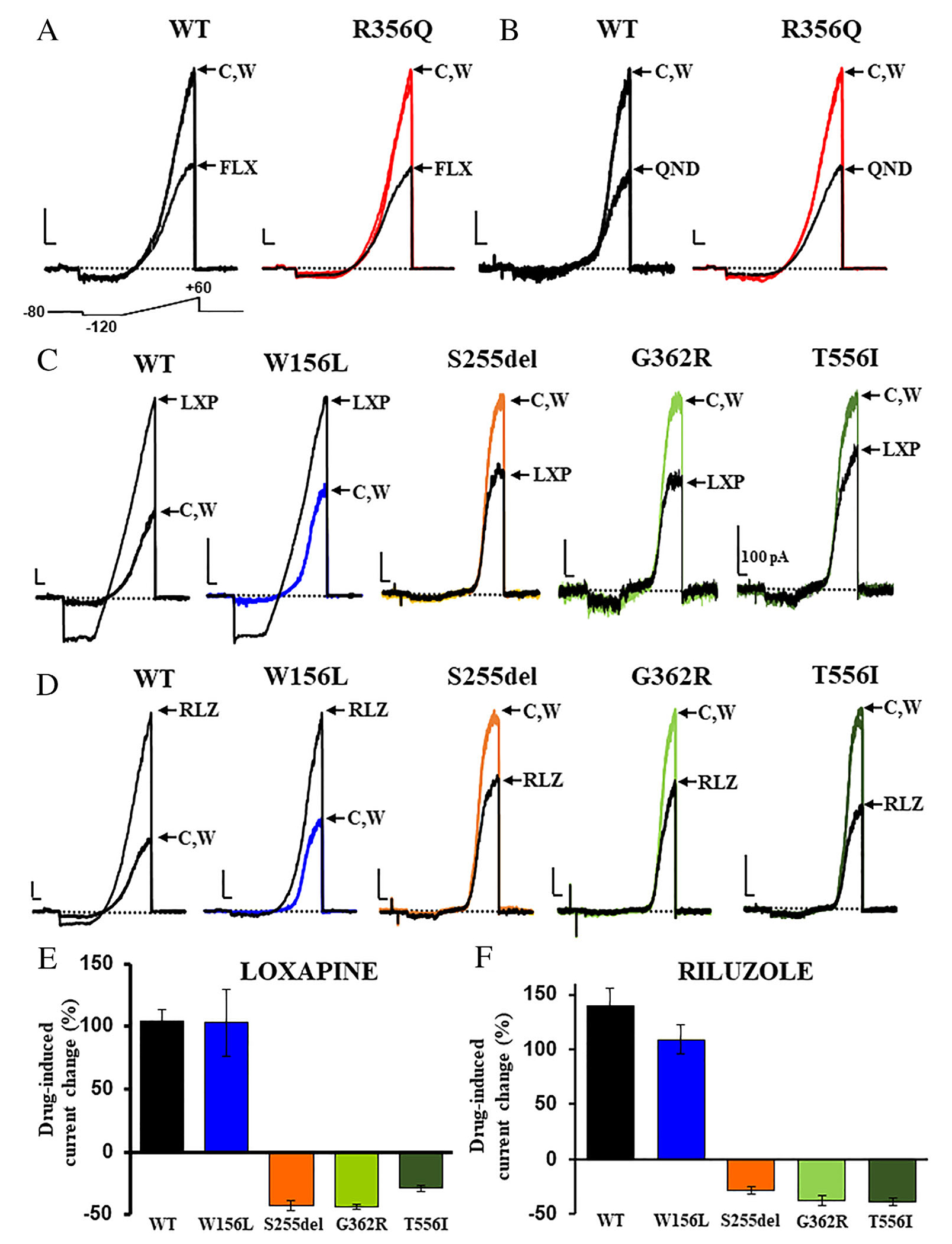
Pharmacological properties of KCNT2 homomeric channels incorporating the newly-identified variants. (A–D) Representative current traces recorded from HEK cells expressing the indicated KCNT2 channels upon exposure to the ramp voltage protocol shown below the first group of traces in control solution (C), after 1–3 minute exposure to 10 μM fluoxetine (FLX; panel A), 100 μM quinidine (QND; panel B), 10 μM loxapine (LXP; panel C), 100 μM riluzole (RLZ; panel D), or upon drug washout (W). Current scale: 200 pA (when different, the value is specified in the figure); time scale: 200 msec. (E, F) Average current densities at +60 mV measured in cells expressing the indicated channels upon exposure to 10 μM loxapine (panel E) or 100 μM riluzole (panel F). [Color figure can be viewed at www.annalsofneurology.org ]

To identify potential pharmacological approaches able to restore channel function, we investigated the *in vitro* sensitivity of KCNT2 channels carrying GoF and LoF variants to potential blockers and activators, respectively. To this aim, we assessed the effects of quinidine (QND) and fluoxetine (FLX), 2 blockers of various potassium channels^30^in KCNT2-GoF channels, and of loxapine (LXP) and riluzole (RLZ), 2 known activators of KCNT-type channels^31^ in KCNT2-LoF channels. Drug concentrations close to the IC_50_ (for blockers) and EC_50_ (for activators) values found in wild-type KCNT2 channels were used. Quinidine (100 μM) and fluoxetine (10 μM) blocked currents carried by KCNT2 channels by about 60% and 45%, respectively (Fig. 5A,B; Table 4); KCNT2 current blockade by both drugs was fully reversible upon drug washout from the recording chamber. Qualitatively similar blocking effects were exerted by both drugs in channels carrying the F240C, R356Q (Fig. 5A,B, respectively), K366E, N750S, or F827L GoF variants herein found in newly-identified patients, as well as those carrying the previously-described Q198E, Y331N, and L880M variants; in KCNT2 N750S or F827L channels, quinidine, but not fluoxetine, blocking efficacy was slightly reduced or increased, respectively (Table 4).

Loxapine (10 μM) and riluzole (100 μM) markedly activated wild-type KCNT2 channels, as well as KCNT2 channels carrying the LoF variants W156L or N182I (Fig. 5C,D, respectively; Table 4). Unexpectedly, both loxapine and riluzole inhibited rather than potentiated the currents carried by channels incorporating the other 3 LoF variants (S255del, G362R, and T556I) found in newly-identified patients, as well as the T242N variant occurring in the patient previously described by Inuzuka et al.^17^ (Fig. 5C,D; Table 4). Average data showing the differential effect of loxapine and riluzole on LoF *KCNT2* variants are shown in Fig. 5E,F, respectively. Altogether, the present results indicate that, while currents from all KCNT2 channels carrying GoF variants, similarly to wild-type KCNT2 channels, were inhibited by quinidine and fluoxetine, KCNT2 channels carrying LoF variants exhibited a characteristic and unpredictable sensitivity to drugs acting as activators in wild-type KCNT2 channels, with some variants showing potentiation, and others displaying a unique blockade by both loxapine and riluzole. To further explore this unique pharmacological response to loxapine and riluzole observed in KCNT2 T242N, S255del, G362R, and T556I channels, and considering the heterozygous state of patients carrying *KCNT2* pathogenic variants, wild-type KCNT2 and KCNT2 T242N, S255del, G362R, or T556I cDNAs were co-transfected in HEK-293 cells at a 0.5:0.5 ratio. Current density in KCNT2 + KCNT2 T242N−, KCNT2 + KCNT2 S255del−, KCNT2 + KCNT2 G362R−, or KCNT2 + KCNT2 T556I-expressing cells was still significantly lower than that recorded in wild-type *KCNT2*-expressing cells, thus displaying functional properties roughly intermediate between homomeric wild-type and mutant KCNT2 channels (Table 4). However, loxapine and riluzole significantly potentiated the currents carried by KCNT2 + KCNT2 S255del−, KCNT2 + KCNT2 G362R−, or KCNT2 + KCNT2 T556I-expressing cells, although the efficacy of both drugs in KCNT2 + KCNT2 G362R channels was still lower than that shown in wild-type KCNT2 channels. Instead, currents from KCNT2 + KCNT2 T242N-expressing cells were blocked by loxapine but were significantly potentiated by riluzole (Table 4), although with reduced efficacy when compared to wild-type KCNT2 channels. These data suggest that each mutant KCNT2 subunit exerts a variable and specific contribution to the pharmacological properties when incorporated in heteromeric channels with wild-type KCNT2 subunits.

## Discussion

### Genetic Landscape and Functional Properties of KCNT2 Variants

Ten pathogenic variants, 9 never reported before, were found in the newly-identified patients. Previous functional studies in *KCNT2* variants showed: (1) a “change-of-function” resulting in an altered channel selectivity with increased Na^+^ permeability (F240L)^16^; (2) GoF properties (R190H and R190P)^14,18^; (3) LoF properties (L48Qfs43* and K564*).^19^ This functional heterogeneity *in vitro* was confirmed and expanded by our results; when expressed in HEK-293 cells, 5 (F240C, R356Q, K366E, N750S, and F827L) out of the 9 novel variants displayed GoF features, and 4 (W156L, S225del, G362R, and T556I) displayed functional properties indicative of a LoF in vitro phenotype. Qualitatively similar functional effects were observed when selected LoF or GoF variants were also expressed in neuron-like RA-differentiated human neuroblastoma SH-SY5Y cells. Moreover, 3 of the 5 previously described but not functionally tested variants (Q198E, Y331N, L880M)^13,15^ displayed in vitro GoF, and 2 (N182I and T242N)^13,17^ LoF features. Thus, KCNT1 and KCNT2 channels, despite their structural and functional similarity, appear to exhibit a remarkable difference in the functional properties of their pathogenic variants responsible for DEEs, with *KCNT1* variants almost invariably promoting a GoF phenotype when assessed *in vitro*, whereas *KCNT2* variants being almost equally distributed between GoF and LoF (10 and 8, respectively).

The occurrence of pathogenic *KCNT2* LoF variants may appear surprising, since the probability of being intolerant to heterozygous LoF mutations, commonly indicated by the pLI scores (https://gnomad.broadinstitute.org/), for both *KCNT1* and *KCNT2* genes is very low (the pLI scores are 0.04 and 0, respectively), suggesting that there is a high incidence of so-called LoF variants in the general population. However, LoF variants used to calculate pLI scores only include non-sense, frameshift, or splice site variants.^23^ Instead, the pLI score should be used with caution when interpreting missense variants, as functional studies (including those herein described) are critically needed to determine whether these will produce GoF or LoF changes at the protein level, and several examples exist in the literature of genes with low pLI scores whose haploinsufficiency-causing variants are clearly pathogenic.^32^ This issue seems even more critical for multimeric proteins, such as K_Na_ channels, as disease-causing variants in few subunits may “poison” the entire tetrameric channel complex and decrease channel function by >50% (dominant-negative effects).^33^ Thus, it seems that the LoF definition captured by the pLI score does not fully reproduce the naturally-occurring genetic complexity, particularly for those genes carrying pathogenic missense variants such as *KCNT2*.

It must be highlighted that mice models of *KCNT2* deletion (Slo2.1KO) show distinct changes in sensory responses (sustained thermal hyperalgesia during inflammatory pain), but no major changes in overall behaviour and growth and no spontaneous seizures.^34,35^ However, all novel *KCNT2* variants herein described are missense, except one which is an in-frame deletion of a single amino acid (S255del). For *KCNT1*, knockout (KO) and knock-in (KI) mice display drastically different phenotypes; in fact, while Kcnt1^−/−^ mice produced deficits in open field behaviour and motor skill learning with only minimal changes in thresholds for electrically and chemically induced seizures, heterozygous KCNT1 KI mice carrying the *KCNT1* R455H variant (homologous to the human R474H GoF variant found in epileptic individuals) had persistent interictal spikes, spontaneous seizures, a substantially decreased threshold for chemically-induced seizure, but no impairment in tasks of exploratory behaviour or procedural motor learning, suggesting that these 2 phenotypes involve different pathways.^36^ Whether similar differences in the phenotypes exist also between *KCNT2* KO and KI animals is currently unknown, as no *KCNT2* KI mouse model has yet been developed which would more faithfully replicate the genetic status of the patients carrying *KCNT2* variants.

### Phenotypic Spectrum and Genotype–Phenotype Correlations

Our novel cohort of 12 patients expands the phenotypic spectrum of *KCNT2*-related disorders, especially toward milder clinical forms. Among the 25 known patients with *KCNT2*-related disorders, 15 (60%) presented with epilepsy. Mean age of epilepsy onset was 3.41 months (range: 1 day-8 months) and 6.25 months (range 2 months-14 months) in GoF and LoF patients, respectively. In our novel series, epilepsy was less common, occurring only in 5/12 (41.67%) of subjects in comparison with 10/13 (77%) patients in the literature, and it was less severe since all newly-described patients were seizure-free at the last follow-up, whereas none of the published patients became seizure-free (Figure 1B). Two novel patients with epilepsy (one harboring a LoF, the other a GoF variant) showed fever sensitivity, not previously described; fever triggered also paroxysmal dystonia episodes in patient #2. Different epilepsy syndromes occurred in patients with GoF or LoF variants. EIMFS was observed in 2 patients for each type of variant; by contrast, WS was reported only in 4 GoF patients (2 with the R190H variant) and in the patient carrying the change-of-function variant. A DEE was reported in 4 patients, 3 GoF (#4, #11, #25) and a LoF (#14), whereas focal epilepsy was diagnosed only in 2 patients both carrying a LoF variant. These findings suggest that WS is related only to GoF variants, while focal epilepsies might be related only to LoF variants; however, due to the limited number of patients and to the heterogeneity of epilepsy syndromes observed, conclusions on genotype–phenotype correlations regarding epilepsy phenotype should be drawn with caution. Regarding the course of epilepsy, in our unpublished cohort, seizures were more frequent at onset and tended to gradually decrease with age until remission. This finding does not replicate the data in the literature, where most of the patients remained drugresistant.

Different degrees of ID/DD were observed in patients carrying GoF or LoF variants. Indeed, a profound/severe ID/DD was observed in 7/12 GoF in whom the information was available and only in 2/7 LoF patients; moderate–severe ID/DD was featured only in 2 GoF patients; moderate ID/DD was reported in 3 GoF and 2 LoF, whereas mild ID/DD was observed only in 2 subjects harboring a LoF variant. The only patient without epilepsy and with normal neurological and developmental status carried a LoF variant. In addition, 7 (4 novel and 3 previously-described) patients were diagnosed with ASD, apparently unrelated with channel functioning alteration, as 4 carried GoF variants, and 3 LoF variants. Overall, the developmental trajectory showed in most cases a delayed acquisition of milestones; however, in few cases (patients #10, #12, #15, #16), a normal or only mildly delayed initial development was followed by a plateauing or regression of the acquisitions at the end of the first or during their second year of life. Interestingly, these latter patients harbored a GoF variant, suggesting that neurodevelopmental regression might be a further distinguishing feature between GoF and LoF variants.

Hypotonia was more commonly observed in GoF patients (10/13, 76.92%) as compared to LoF ones (2/8, 25%).

Similar dysmorphic features (such as pronounced eyebrows, long eyelashes, diastema, and hypertrichosis) were reported in 10/14 patients harboring GoF variants, 7 of them carrying pathogenic variants at the p.190 position, suggesting that these dysmorphisms might be a distinguishing characteristic of GoF variants, particularly those at the p.190 residue.^14,18^ Patient #12 however, carrying a GoF variant, presented only mild and slightly different dysmorphic features (Table 1). Only 2 patients (#5 and #10) with a LoF variant presented dysmorphic features, that differed only in patient #5 from those observed in GoF patients. Interestingly, the dysmorphic features observed in the vast majority of our GoF patients partly resemble those seen in a neurodevelopmental syndrome caused by GoF variants in another potassium channel (*KCNK4*)^37^ and hypertrichosis was also reported in other K^+^ channelopathies such as Cantù syndrome (*ABCC9* and *KCNJ8*) and Zimmermann-Laband syndrome 1 (*KCNH1*) and 3 (*KCNN3*).^38^ Overall, phenotype–genotype correlations, based on the current evidence, suggest that GoF variants were more often associated with severe ID/DD, earlier epilepsy onset, West syndrome, hypotonia, and dysmorphisms as compared to LoF variants which were associated with clinical pictures at the milder end of the phenotypic spectrum with a propensity to language delay (Figure 1C).

### Pharmacological Study and Therapeutic Implications

For patients carrying GoF *KCNT1* variants, sensitivity^9,39^ or insensitivity^40^ to treatment with the potassium channel blocker quinidine parallels drug responses in vitro. Moreover, heterogeneous functional changes (LoF or GoF) triggered by pathogenic *KCNT2* variants in vitro may require distinct pharmacological approaches to restore channel function in vivo.^41^ Our in vitro results revealed that each *KCNT2* variant exhibits a distinct pattern of pharmacological sensitivity. Indeed, while GoF *KCNT2* variants are all blocked by quinidine and fluoxetine, KCNT2 channels carrying W156L or N182I LoF variants, similarly to wild-type KCNT2 channels, were potentiated by loxapine or riluzole, 2 openers of wild-type KCNT1 channels,^31^ showing for the first time their capability to activate also KCNT2 channels. Loxapine or riluzole instead inhibited T242N, S255del, G362R, or T556I LoF KCNT2 currents; this latter pharmacological response is predicted to aggravate variant-induced functional derangement. Notably, incorporation of wild-type KCNT2 subunits into heteromeric channels with S255del, G362R, or T556I mutant subunits switched the response to loxapine and riluzole from inhibitory to activating; a similar inversion (from blocker to activator) also occurred for riluzole in KCNT2/KCNT2 T242N channels, whereas loxapine still blocked these heteromeric channels.

Several mechanisms, including variant-dependent effects on the drug binding site or on the conformational changes translating drug binding into functional changes, as well as the variable contribution of each (wild-type or mutant) subunit to these responses, might explain the unique pharmacological pattern observed. In fact, for modular proteins such as ion channels, it has been proposed that the ability of some allosteric effectors to act as agonists under some conditions, and antagonistic under others does not require that the nature of the interaction between the ligand and its binding site be different, but may be also influenced by changes in the energetic coupling between the different regions of the protein^42^; whether the described missense variants in *KCNT2* directly affect binding of the ligands or the allosteric coupling translating binding into changes in opening probabilities is worth investigating in future studies. Nonetheless, a recent high-throughput in vitro evaluation of epilepsy-associated variants in the potassium channel gene *KCNQ2*^43^ has revealed a similar heterogeneity among variants in their response to the activator retigabine,^44^ with some variants exhibiting little to no response, and others showing effects equal to or greater than wild-type channels. The present results add another layer of complexity when attempting to correlate ion channel gene variants with their in vitro effects and pharmacological sensitivity, and provide support for a standardized algorithm in which functional and pharmacological properties of each pathogenic variant need to be experimentally determined in vitro to enable targeted therapeutic strategies based on the molecular phenotype, as suggested for other potassium channelopathies.^45^

In our cohorts, no ASM emerged as being more effective. Quinidine has been used with mixed results in patients featuring *KCNT1* variants,^9,46,47^ and in 1 *KCNT2* patient, with positive effects despite dosing limitations due to QTc prolongation.^14^ Quinidine showed a dramatic effect on seizure frequency leading to seizure freedom in patient #7 (in combination with ketogenic diet and cannabidiol) 1 month after initiation. Although it cannot be excluded that the other therapeutic measures implemented shortly before may have contributed to seizure freedom, quinidine appeared temporally as the critical element leading to seizure cessation. However, quinidine cannot be recommended in all cases of *KCNT2*-related diseases, even more so in the absence of functional data assessing whether the variant has GoF or LoF effects, given their association with somewhat overlapping clinical pictures. Moreover, quinidine has so far only been tried in 2 patients with *KCNT2* variants, and larger number of patients would be needed to draw conclusions on its effectiveness. On the other hand, fluoxetine or loxapine/riluzole were not tested in our newly-described patients with epilepsy since they were all seizure-free at time of inclusion.

### Study Limitations and Conclusions

This study, which includes the largest case series reported to date for *KCNT2* and comprises functional and pharmacological analyses for all untested variants reported so far, expands the genotype and phenotype spectra for *KCNT2*-associated disorders, and highlights novel genotype–phenotype associations which may facilitate timely diagnosis and counseling. Our findings suggest that *KCNT2* variants should be considered in patients with early-onset epilepsy or isolated neurodevelopmental disorders. Nonetheless, our study has several limitations. The first is common to most studies in rare epilepsies^46^ (and rare diseases in general) and refers to the heterogeneity of clinical data reporting, especially for the published cases, with regards to electroclinical, neuropsychological, psychiatric, and drug response assessment which were not performed systematically in all patients. Another limitation concerns the functional assessment of pathogenic variants, which has been only performed in in vitro heterologous expression systems; both GoF and LoF functional phenotypes were observed, with a roughly 1:1 ratio, a remarkable difference when compared to the homologous *KCNT1* gene whose pathogenic variants are almost invariably GoF. However, additional studies *in vivo* or in *in vitro* models more closely reproducing the patient’s genetic background (such as from human neurons derived from induced pluripotent stem cells) should be performed to unequivocally prove, rather than suggest, LoF effects on KCNT2 currents as a novel disease pathogenetic mechanism. Such studies will also clarify whether GoF and LoF variants in KCNT2 prompt distinct dis-homeostatic mechanisms and require distinct pharmacological approaches.^29^

## Supplementary Material

Supplementary Figure S1

Supplementary table S1-2-3

Additional supporting information can be found in the online version of this article.

## Figures and Tables

**TABLE 1.** Novel Cohort, Demographic, Genetic, and Clinical Data

Pts	Pt 1	Pt 2	Pt 3	Pt 4	Pt 5	Pt 6	Pt 7	Pt 8	Pt 9	Pt 10	Pt 11	Pt 12
Sex	M	F	M	F	F	F	F	F	M	M	M	M
Age	10 yr	5 yr	6 yr	13.5 yr	10 yr	40 yr	22 mo	5 yr	6 yr	6 yr 5 mo	14 yr	22 yr
cDNA	c.467G > T	c.569G > A	c.569G > A	c.719T > G	c.763_765del	c.763_765del	c.1067G > A	c.1084G > C	c.1096A > G	c.1667C > T	c.2249A > G	c.2479 T > C
Prot. ch.	W156L	R190H	R190H	F240 C	S255 del	S255 del	R356Q	G362R	K366 E	T556 I	N750 S	F827 L
Inherit.	N.A.	pat.^a^	de novo	de novo	mat.	N.A.	de novo	de novo	de novo	de novo	de novo	de novo
Funct.	LoF	GoF	GoF	GoF	LoF	LoF	GoF	LoF	GoF	LoF	GoF	GoF
ID/DD	mild	mod	severe	mod-severe	mod	mild	not appl.	no	mod-severe	mod	severe	mod
Lang.	first words 17 mo	70 words	<10 words	few words	10 words	delay	1–2 words	norm.	50 words	simp. sent.	simp. sent.	simp. sent.
Autism	no	no	no	no	no	no	not appl.	yes	yes	yes	yes	no
Behav Dis.	HA, AD	no	HA	anx	anx, AB	AB	not appl.	no	no	AB, HA	yes	yes
Neuro Feat.	Bab., weakn lower limbs, paraso	hyp, parox. dyst., arouse	mild hyp	no	no	no	hyp	no	hyp, weakn lower limbs, gait ataxia, arouse	ataxia clums, poor motor skills	hyp, ataxia	no
Dysm. Feat.	no	diast., curved eyebr., long eyelas, short philtr., full lips, hypert	large eyebr., long eyelas., hypert, brachy	round nasal tip, short philtr., full lips, thick eyebr, long eyelas, hypert	↑IOD, flat nasal root, round tip, brachy, clino, synd	no	no	no	no	long eyelas, short philtr, full upper lip, tooth shift	no	ear malf., campt, clino
Other	const.	B12 deficit	asth.	↓HC	astigm.	/	/	/	/	joint ↑ mobil.	equin. foot, astigm ↓HC	/

Abbreviations: AB = aggressive behaviour, AD = attention deficit, anx.: anxiety, appl. = applicable, arouse = arousals, asth. = asthma, astigm = astigmatism, Bab. = Babinski sign, Behav. Dis = Behavioral disorders, brachy = brachydactyly, campt = camptodactyly, clino = clinodactyly, const = constipation, diast = diastema, DD = developmental delay, dysm = dysmorphic, equin. = equinus, eyebr.: eyebrows, eyelas = eyelashes, F = female, Funct = Functional Properties, GoF = gain of function, HA = hyperactivity, ↓HC = reduced head circumference (microcephaly), hyp = hypotonia, hypert = hypertrichosis, ID = intellectual disability, ↑IOD = increased intra-ocular distance (hypertelorism), inherit. = inheritance, LoF = loss of function, M = male, malf. = malformation, mat. = maternal, mo = months, ↑ mobil. = increased mobility, mod = moderate, N.A. = not available, Neuro = neurological, paraso = parasomnias, pat. = paternal, philtr. = philtrum, Prot. Ch. = protein change, sent. = sentences, simp = simple, synd = syndactyly, unrem = unremarkable, weakn = weakness.

a Father = somatic mosaicism 15%.

**TABLE 2.** Novel Cohort, Epilepsy Features, EEG, and Neuroimaging Findings

Pts	Pt 1	Pt 2	Pt 3	Pt 4	Pt 5	Pt 6	Pt 7	Pt 8	Pt 9	Pt 10	Pt 11	Pt 12
cDNA	c.467G > T	c.569G > A	c.569G > A	c.719T > G	c.763_765del	c.763_765del	c.1067G > A	c.1084G > C	c.1096A > G	c.1667C > T	c.2249A > G	c.2479 T > C
Prot. ch.	W156L	R190H	R190H	F240 C	S255 del	S255 del	R356Q	G362R	K366E	T556 I	N750 S	F827 L
Funct.	LoF	GoF	GoF	GoF	LoF	LoF	GoF	LoF	GoF	LoF	GoF	GoF
Epilepsy	yes	no	yes	yes	no	no	yes	no	no	no	yes	no
Age sz onset	14 mo	/	6 mo	1 mo	/	/	4,5 mo	/	/	/	6 mo	/
Sz (onset)	F motor, FBTC	/	spasm	GTC	/	/	spasm	/	/	/	FS	/
Seizures (f-up)	F motor, FBTC	/	spasm	GTC	/	/	C, M, motor	/	/	/	FS, GTC	/
Epilepsy type	Focal	/	West	DEE	/	/	West → DEE	/	/	/	DEE	/
Current Treat.	VPA	/	no ASM	no ASM	/	/	KD, CBD, Quinid	/	/	/	Sulth., KD	/
Seizure outcome (age achieved)	sz free (10 y)	/	sz free (1y 1mo)	sz free (4 mo)	/	/	sz free (9 mo)	/	/	/	sz free (10 y)	/
EEG	bi-Fr SW	norm.	hyps, poly-SW	norm.	N.A.	N.A.	hyps, now norm.	N.A.	Sh-W FrC > R	multiF Sh-W	F S CPT-L	N.A.
Brain MRI	norm	mild pachygyria	mild volume loss	norm.	norm.	N.A.	norm.	N.A.	norm.	N.A.	norm.	N.A.

Abbreviations: ASM = anti-seizure medication, Bi-Fr = bilateral frontal, C = central, CBZ = carbamazepine, F = focal, FBTC = focal to bilateral tonic–clonic, Fr = frontal, FS = febrile seizures, Funct = functional properties, GoF = = gain of function, GTC = bilateral tonic clonic with generalized onset, hyps = hypsarrhythmia, KD = ketogenic diet, L = left, LoF = loss of function, mo = months, multiF = multifocal, N.A. = not available, norm. = normal, P = parietal, Quinid = quinidine, R = right, S = spikes, sp = spasms, Sh-W = sharp wave, Sulth = sulthiame, SW = spike and wave, Sz = seizure, T: temporal, Treat. = treatment, VPA = valproate.

**TABLE 3.** Biophysical Properties of KCNT2 Channels Carrying the Indicated Variants

	Biopysical Properties					
	*n*	Current Density at + 60 mV (pA/pF)	V_½_ (mV)	*k* (mV/*e*fold)	I_INST_/I_SS_	Functional Effects
NT	4	1.3 ± 0.2	–	–	–	–
KCNT2(WT)	52	55.3 ± 2.2	−9.6 ± 1.4	18.3 ± 0.8	0.52 ± 0.02	–
New variants						
W156L	44	40.6 ± 2.8*	−11.9 ± 1.3	12.6 ± 0.8*	0.50 ± 0.02	LoF
F240C	17	47.4 ± 5.0	−22.1 ± 1.3*	10.2 ± 0.9*	0.48 ± 0.03	GoF
S255del	12	24.9 ± 2.6*	−13.5 ± 2.2	10.7 ± 1.5*	0.49 ± 0.05	LoF
R356Q	16	125.6 ± 20.3*	−16.4 ± 1.5*	17.4 ± 1.0	0.55 ± 0.03	GoF
G362R	26	24.2 ± 1.7*	−12.8 ± 1.2	9.5 ± 0.8*	0.46 ± 0.03	LoF
K366E	38	279.3 ± 20.7*	−35.3 ± 1.7*	28.2 ± 1.1*	0.79 ± 0.02*	GoF
T556I	13	31.2 ± 1.9*	−12.8 ± 3.1	14.8 ± 1.8	0.43 ± 0.02*	LoF
N750S	12	122.5 ± 12.4*	−6.0 ± 2.0	22.8 ± 1.2	0.59 ± 0.03	GoF
F827L	15	192.9 ± 23.5*	−23.2 ± 2.4*	23.8 ± 1.5*	0.58 ± 0.02*	GoF
WT + S255del	14	35.3 ± 3.5**	−11.6 ± 2.2	13.8 ± 1.6*	0.41 ± 0.04*	LoF
WT + G362R	18	41.1 ± 3.0**	−11.9 ± 2.1	16.2 ± 1.4	0.55 ± 0.04	LoF
WT + T556I	28	41.9 ± 3.4**	−14.8 ± 1.5	12.7 ± 1.0*	0.48 ± 0.03	LoF
Known variants						
N182I	10	34.4 ± 2.9*	−12.4 ± 1.4	10.7 ± 1.1*	0.47 ± 0.05	LoF
R190H†	49	147.5 ± 13.6*	−14.9 ± 1.8*	24.5 ± 1.0*	0.77 ± 0.02*	GoF
R190P†	24	398.3 ± 45.5*	−48.0 ± 2.3*	28.6 ± 1.6*	0.93 ± 0.01*	GoF
Q198E	16	257.5 ± 35.0*	−31.0 ± 2.2*	24.2 ± 1.4*	0.79 ± 0.03*	GoF
T242N	12	24.8 ± 1.6*	−12.0 ± 2.3	11.4 ± 1.3*	0.41 ± 0.05*	LoF
Y331N	21	117.4 ± 18.5*	−20.7 ± 2.6*	22.7 ± 1.4	0.56 ± 0.04	GoF
L880M	32	68.4 ± 7.5*	−14.0 ± 1.3*	15.4 ± 0.9	0.42 ± 0.02*	GoF
WT + T242N	16	36.4 ± 2.9**	−10.1 ± 2.8	16.2 ± 1.5	0.38 ± 0.03*	LoF
VUS						
R47K	11	50.3 ± 7.6	−10.6 ± 1.2	10.9 ± 0.8*	0.36 ± 0.02*	–
L313S	19	62.0 ± 7.2	−12.7 ± 1.8	15.6 ± 1.3	0.51 ± 0.02	–
T910M	20	61.5 ± 7.9	−11.8 ± 2.2	22.1 ± 1.4	0.55 ± 0.04	–

* *p* < 0.05 versus KCNT2;

** *p* < 0.05 versus respective homomers;

† Data already reported in ref.^14^

**TABLE 4.** Pharmacological Properties of KCNT2 Channels Carrying the Indicated Variants

	Pharmacological Properties				
	*n*	100 μM QND-Induced Current Change (% of CTL)	10 μM FLX-Induced Current Change (% of CTL)	10 μM LXP-Induced Current Change (% of CTL)	100 μM RLZ-Induced Current Change (% of CTL)
KCNT2 (WT)	26	−59.5 ± 3.0	−44.5 ± 1.8	+106.0 ± 9.6	+142.1 ± 17.2
New variants					
W156L	15	–	–	+103.0 ± 26.5	+108.9 ± 13.1
F240C	19	−64.0 ± 3.7	−71.5 ± 5.0*	–	–
S255del	15	–	-	−44.0 ± 1.9*	−28.2 ± 3.7*
R356Q	19	−55.0 ± 3.5	−52.5 ± 1.7	–	–
G362R	13	–	–	−42.7 ± 3.7*	−37.6 ± 4.5*
K366E	21	−54.7 ± 3.1	−56.4 ± 2.7	-	-
T556I	13	–	–	−29.0 ± 2.8*	−38.2 ± 3.6*
N750S	18	−24.4 ± 6.3*	−50.5 ± 1.4	–	–
F827L	18	−70.3 ± 1.6*	−37.9 ± 1.8	–	–
WT + S255del	14	–	–	+76.2 ± 13.4	+94.9 ± 8.1
WT + G362R	13	–	–	+52.8 ± 10.7*	+61.5 ± 3.8*
WT + T556I	13	–	–	+89.3 ± 19.5	+111.3 ± 16.2
Known variants					
N182I	12	–	–	+111.7 ± 11.6	+102.2 ± 8.8
R190H†	17	−67.1 ± 3.8†	−45.3 ± 2.8	–	–
R190P†	15	−75.3 ± 4.8*,†	−55.2 ± 3.9	–	–
Q198E	15	−46.4 ± 2.5	−50.5 ± 0.8	–	–
T242N	13	–	–	−39.2 ± 3.1*	−26.7 ± 1.5*
Y331N	17	−55.8 ± 4.2	−54.1 ± 1.4	–	–
L880M	17	−55.4 ± 4.3	−50.5 ± 2.5	–	–
WT + T242N	16	–	–	−30.8 ± 2.2*	+52.4 ± 9.0*

* *p* < 0.05 versus KCNT2;

** *p* < 0.05 versus respective homomers;

† Data already reported in ref.^14^

## Data Availability

All main results of this study are available within the article and its Supplementary material. Further inquiries can be directed to the corresponding authors.
