# Supplementary figures and images for: KCNT2-Related Disorders: Phenotypes, Functional, and Pharmacological Properties
Source: Ann Neurol. Author manuscript; Available in PMC 2026 Mar 23. (PMC13007567; doi:10.1002/ana.26662)

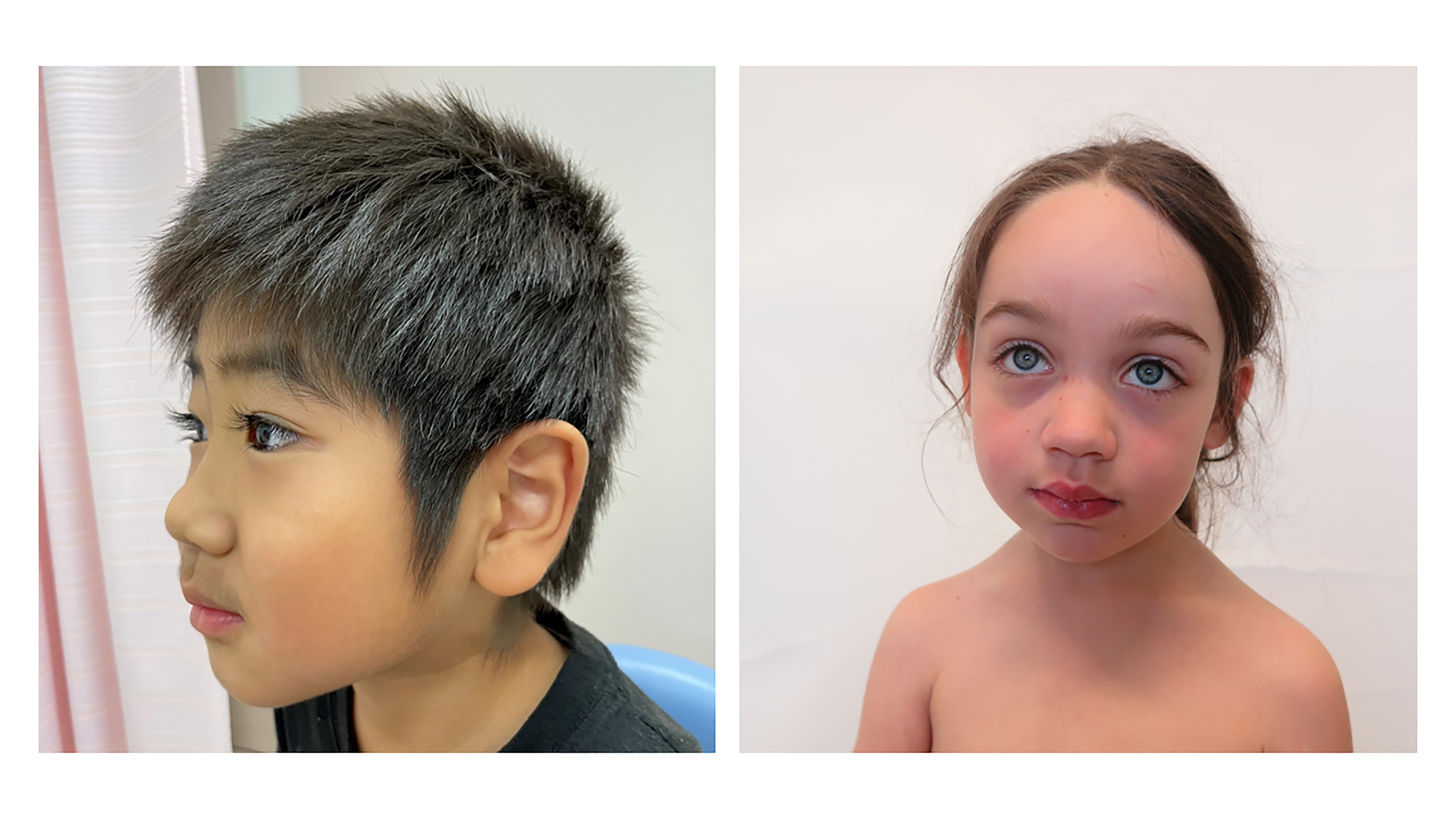

Supplement: Supplementary Figure S1 [file NIHMS2144890-supplement-Supplementary_Figure_S1.tif]
