# Supplementary material for: KCNT2-Related Disorders: Phenotypes, Functional, and Pharmacological Properties
Source: Ann Neurol. Author manuscript; Available in PMC 2026 Mar 23. (PMC13007567; doi:10.1002/ana.26662)
Supplement: Supplementary table S1-2-3 [file NIHMS2144890-supplement-Supplementary_table_S1-2-3.docx]

**Supplementary table 1: *KCNT2* study group members and affiliations**

| AUTHOR NAME AND TITLE | Affiliations |
| --- | --- |
| Ilenio Servettini PhD | Department of Medicine and Health Sciences “Vincenzo Tiberio”, University of Molise, Campobasso, Italy; Department of Neuroscience, University of Naples “Federico II”, Naples, Italy |
| Giorgio Belperio, MBBS | Dept. of Science and Technology, University of Sannio, Benevento, Italy |
| Erik-Jan Kamsteeg PhD | Department of Human Genetics, Radboudumc, Nijmegen, The Netherlands |
| Koji Takahashi MD | Department of Pediatrics, Tsuchiura Kyodo General Hospital, Tsuchiura, Ibaraki 300-0028, Japan |
| Satomi Mitsuhashi MD, PhD | Department of Human Genetics, Yokohama City University Graduate School of Medicine, Yokohama, Kanagawa 236-0004, Japan; Department of Genomic Function and Diversity, Tokyo Medical and Dental University, Tokyo, Japan |
| Elizabeth E. Palmer MBBS, PhD | Sydney Children’s Hospitals Network-Randwick, UNSW, Sydney, Australia; School of Paediatric and Child Health, Faculty of Clinical Medicine and HealthUNSW, Sydney, Australia. |
| Ann M. Bye MBBS, PhD | Sydney Children’s Hospitals Network-Randwick, UNSW, Sydney, Australia; School of Paediatric and Child Health, Faculty of Clinical Medicine and HealthUNSW, Sydney, Australia. |
| Irene Madrigal PhD | Biochemistry and Molecular Genetics Department, Hospital Clinic of Barcelona, IDIBAPS (Institut de Investigacions Biomèdiques August Pi I Sunyer); CIBER of Rare Diseases (CIBERER), Barcelona, Spain |
| Maria Isabel Alvarez-Mora PhD | Biochemistry and Molecular Genetics Department, Hospital Clinic of Barcelona, IDIBAPS (Institut de Investigacions Biomèdiques August Pi I Sunyer); CIBER of Rare Diseases (CIBERER), Barcelona, Spain |
| Aurora Sánchez MD | Biochemistry and Molecular Genetics Department, Hospital Clinic of Barcelona, IDIBAPS (Institut de Investigacions Biomèdiques August Pi I Sunyer); CIBER of Rare Diseases (CIBERER), Barcelona, Spain |
| Stefano Meletti MD, PhD | Department of Biomedical, Metabolic, and Neural Science, University of Modena and Reggio Emilia, Modena, Italy; Neurology Unit, University Hospital, OCB, Modena, Italy. |
| Ingo Helbig MD | Department of Neuropediatrics, Christian-Albrechts-University Kiel & University Hospital Schleswig-Holstein, Campus Kiel, Germany; Institute of Clinical Molecular Biology, Christian-Albrechts-University of Kiel, Kiel, Germany; Division of Neurology, Children’s Hospital of Philadelphia, Philadelphia, USA; The Epilepsy NeuroGenetics Initiative (ENGIN), Children's Hospital of Philadelphia, Philadelphia, USA; Department of Biomedical and Health Informatics (DBHi), Children’s Hospital of Philadelphia, Philadelphia, USA; Department of Neurology, University of Pennsylvania, Perelman School of Medicine, Philadelphia, USA |
| Pauline Le Tanno MD | Department of Medical Genetics, Grenoble-Alps University Hospital, Grenoble, France |
| Bénédicte Gerard PharmD, PhD | Laboratoires de Diagnostic Génétique, Unité de génétique moléculaire, Nouvel Hôpital Civil, IGMA, Strasbourg Cedex, France |
| Salima El Chehadeh MD | Service de Génétique Médicale, Institut de Génétique Médicale d’Alsace (IGMA), Hôpitaux Universitaires de Strasbourg, Strasbourg, France; Institut de Génétique et de Biologie Moléculaire et Cellulaire (IGBMC), INSERM U1258, CNRS-UMR7104, Université de Strasbourg, Illkirch Graffenstaden, France; Laboratoire de Génétique Médicale, UMRS_1112, Institut de Génétique Médicale d’Alsace (IGMA), Université de Strasbourg et INSERM, Strasbourg, France |

**Supplementary table 2: Literature cohort**

| **Pts** | **Pt 13**  Ref^19^ (ptB) | **Pt 14**  Ref^13^(pt#1) | **Pt 15**  Ref^14^ (pt#1) | **Pt 16**  Ref^18^ (pt#2) | **Pt 17** Ref^20^(pt#29) | **Pt 18**  Ref^14^ pt#2) | **Pt19** Ref^18^(pt#1) | **Pt 20**  Ref^15^ (pt#2) | **Pt 21**  Ref^16^ | **Pt 22** Ref^17^ | **Pt 23**  Ref^15^ (pt#1) | **Pt 24**  Ref^19^ (pt A) | **Pt 25**  Ref^13^ (pt#2) |
| --- | --- | --- | --- | --- | --- | --- | --- | --- | --- | --- | --- | --- | --- |
| **Sex** | Female | Male | Female | Female | Male | Female | Female | Male | Male | Male | Male | Female | Male |
| **Age** | 29 yrs | 6 yrs | 9 yrs | 5,5 yrs | 6 yrs | 13 yrs | 32 yrs | 9 yrs | 10yrs | 17 yrs | 5 mo | 3 mo | 5 yrs |
| **cDNA** | c.143-144delTA | c.545A>T | c.569G>A | c.569G>A | c.569G>A | c.569G>C | c.569G>C | c.592C>G | c.720T>A | c.725C > A | c.991T>A | c.1690A>T | c.2638C>A |
| **Prot. Ch.** | L48Qfs43* | N182I | R190H | R190H | R190H | R190P | R190P | Q198E | F240L | T242N | Y331N | K564* | L880M |
| **Inherit** | *De novo* | *NA* | *De novo* | *De novo* | *De novo* | *De novo* | *De novo* | *De novo* | *De novo* | *De novo* | *De novo* | *De novo* | *NA* |
| **Funct.** | LoF | LoF | GoF | GoF | GoF | GoF | GoF | GoF | change* | LoF | GoF | LoF | GoF |
| **Epilepsy** | Yes | Yes | Yes | No | No | Yes | No | Yes | Yes | Yes | Yes | Yes | Yes |
| **Age sz onset** | 4 mo | N.A. | 8 mo | / | / | 1 day | / | 1,5 mo | 3 mo | 5 mo | 8 days | 2 mo | N.A. |
| **Sz (onset)** | F migr | N.A. | sp | / | / | GTC | / | F migr | staring | To | spasms | EM, To | N.A. |
| **Sz types** | F migr. | N.A. | To, GTC | / | / | GTC, A | / | F migr | sp, To, M, EM, AA,GTC | hyperkin F | spasms | EM, To | N.A. |
| **Epilepsy type** | EIMFS | DEE | West -> LG | / | / | EIMFS | / | EIMFS like | West -> DEE | Focal | Ohtahara -> West | EIMFS | DEE |
| **Treatm.** | N.A. | N.A. | Sulth, VPA, VGB, TPM, LEV, CLB, GBP,LTG,RFM, MP, KD,Quin. | / | / | PB - now suspended | / | VPA, TPM, LTG, NZP | TPM, NRZ, LEV, LTG, VGB, ESM, ZNS, VPA, KD, UKISS | CBZ, LEV and OXC, other ASMs | PB, TPM | VPA, LTG and LEV; other ASMs | N.A. |
| **Sz outcome** | N.A. | DR | DR | / | / | no ASMs | / | DR | DR | DR | DR | DR | DR |
| **EEG** | N.A. | Sh-W and slow Fr-T R | Hyps -> Sh-slow-W, gen slow -> bil S | N.A. | bil T and F epi | Gen/multiF epi; sz migr | NA | MultiF epi, gen epi | multiF epi, hyps | multiF epi; sz onset ant. bil. | burst suppression -> hyps | slow bg, multiF epi; sz migrating | normal |
| **ID/DD** | Delayed | Profound | Severe | Moderate | Severe | Severe | Severe | Profound | Profound | Severe | Not applic. | Not applic. | Delayed |
| **Language** | N.A. | Non-verbal | Non-verbal | Few words | 6-7 words | 12 words | Sentences | Non-verbal | Non-verbal | Non-verbal | Not applic. | Not applic. | N.A. |
| **Autism** | N.A. | No | No | Yes | Yes | N.A. | N.A. | N.A. | No | yes | not applic | N.A. | N.A. |
| **Behav.** | N.A. | N.A. | N.A. | HA and AB | AD, repetit. | AB | N.A. | N.A. | No | N.A. | not applic. | N.A. | N.A. |
| **Neurological Features** | N.A. | Hy, unable to walk | Hy | hy, sleep disorder, falls | N.A. | Hy | Hy | regression, imp walking | Hy, poor VA, imp. walking, insomnia | Spasticity,ataxia | hy, poor VA | Hy, limited VA | N.A. |
| **Dysmor.** | N.A | N.A. | Prominent eyebrows, long eyelashes, short philtrum, diastema, hypertrichosis | prominent eyebrows, long lashes, spaced teeth, hypertrichosis | elongated face, broadbased nose, short filter, prognathism | Prominent eyebrows, long eyelashes, short philtrum, diastema, hypertrichosis | synophrys, long eyelashes, diastema, hypertrichosis | Prominent eyebrows, long eyelashes, short philtrum, hirsutism | No | N.A. | Prominent eyebrows, long eyelashes, short philtrum, hirsutism | N.A. | N.A. |
| **Other** |  | EMG alterations |  | constipation, wheezing | Bruxism | Self-harm | PO, IFG |  |  | startles |  |  |  |
| **Brain MRI** | N.A. | thin CC, dilat lat. Vn. | Atrophy, delayed myel. | normal | N.A. | normal | CT normal | Normal | ↓WM, thin CC | normal | normal | normal | N.A. |

Abbreviations: A: absences, AA: atypical absences, AB: aggressive behaviour, ACTH: corticotropin, AD: attention deficit, applic: applicable, ASM: anti-seizure medication, bg: background activity bil: bilateral, Behav: behavioural disorders, Bi-Fr: bilateral frontal, C: central, CC: corpus callosum, CBZ: carbamazepine, CLB: Clobazam, DD: developmental delay, disorg: disorganized, DR: drug-resistant, Dysmor: dysmorphic features, EM: eyelid myoclonia, epi: epileptic activity, ESM: Ethosuximide, F: focal, FBTC: focal to bilateral tonic clonic, Fr: frontal, FS: febrile seizures, gen: generalized, Funct: functional properties, GBP: gabapentin, GoF: gain of function, GTC: bilateral tonic clonic with generalized onset, HA: hyperactivity, Hy: hypotonia, Hyperkin: hyperkinetic, hyps: hypsarrhythmia, ID: intellectual disability, IFG: impaired fasting glucose, Inherit.: inheritance, KD: ketogenic diet, L: left, LEV: Levetiracetam, LoF: loss of function, LTG: Lamotrigine, M: myoclonic, migr: migrating, mo: months, MP: Methylprednisolone, NRZ: Nitrazepam, OXC: Oxacarbazepine, P: parietal, PB: phenobarbital, PO: polycystic ovary, PP: Pyridoxal Phosphate, Prot. Ch.: protein change, Py: pyramidal signs, Quin: quindine, R: right, repet: repetitive behaviour, RFM: Rufinamide, S: spike, Sh-W: sharp wave, sp: spasms, SW: spike and wave, Sulth: Sulthiame, Sz: seizure, T:temporal, To: tonic, TPM: Topiramate, Treatm: treatment, VA: visual attention, VGB: vigabatrin, VPA: valproate, ZNS: Zonisamide. *Change of function: loss of K+ specificity and permeability to other cations, ↓: reduced.

**Supplementary Table 3.** Inter- and intra-subunit interactions identified for each of the indicated residues in the closed and open configurations of KCNT2 subunits. Top: interactions identified when the respective position is occupied by the amino acid occurring in the wild-type KCNT2 channel. Bottom: interactions identified when the respective position is occupied by the amino acid substituted in each KCNT2 variant.

|  |  |  | **Intra-subunits interactions** | | | **Inter-subunits interactions** | | |
| --- | --- | --- | --- | --- | --- | --- | --- | --- |
|  |  | **Closed State** | | **Open State** | **Closed State** | | **Open State** |  |
| **Novel variants**  **(Tables 1 and 2)** | **W156** | L159 | | none | none | | none |  |
|  | **F240** | L209, C213, T239, A266 | | L209, C213, C236, A266 | none | | none |  |
|  | **S255** | E221, T252 | | E221, T252 | none | | none |  |
|  | **R356** | Y322, Q328, Y331, Q355 | | Y322, W353 | none | | none |  |
|  | **G362** | none | | none | none | | none |  |
|  | **K366** | none | | none | D812 | | none |  |
|  | **T556** | S561 | | none | none | | none |  |
|  | **N750** | E748 | | K719 | none | | none |  |
|  | **F827** | Q849 | | none | none | | none |  |
| **Published variants**  **(Supplementary Table 2)** | **N182** | E178 | | E178, H185 | none | | Q349 |  |
|  | **Q198** | none | | none | none | | none |  |
|  | **T242** | S241 | | none | T242 | | T242 |  |
|  | **Y331** | E299, R356 | | E299 | none | | K128, E133 |  |
|  | **L880** | L1080 | | L1080 | none | | none |  |

|  |  |  | **Intra-subunits interactions** | | **Inter-subunits interactions** | |
| --- | --- | --- | --- | --- | --- | --- |
|  |  | **Functional effect** | **Closed State** | **Open State** | **Closed State** | **Open State** |
| **Novel variants**  **(Tables 1 and 2)** | **W156L** | **LoF** | L159 | L159 | none | none |
|  | **F240C** | **GoF** | A266 | A266, L270 | none | none |
|  | **S255del** | **LoF** | -- | -- | -- | -- |
|  | **R356Q** | **GoF** | Y322 | Y322 | none | none |
|  | **G362R** | **LoF** | D369 | D369 | none | none |
|  | **K366E** | **GoF** | R190 | none | none | none |
|  | **T556I** | **LoF** | none | none | none | F37 |
|  | **N750S** | **GoF** | K719 | K719 | none | none |
|  | **F827L** | **GoF** | V823, I835 | V823, I835 | none | none |
| **Published variants**  **(Supplementary**  **Table 2)** | **N182I** | **LoF** | H185 | none | P351 | I350, P351 |
|  | **Q198E** | **GoF** | none | none | none | R346 |
|  | **T242N** | **LoF** | none | none | none | none |
|  | **Y331N** | **GoF** | none | Y291, E299 | none | none |
|  | **L880M** | **GoF** | none | W477, L860, L1080 | none | none |
